# Patterns of Occurrence and Activity of Entomopathogenic Fungi in the Algarve (Portugal) Using Different Isolation Methods

**DOI:** 10.3390/insects11060352

**Published:** 2020-06-04

**Authors:** Francisco Ángel Bueno-Pallero, Rubén Blanco-Pérez, Ignacio Vicente-Díez, José Antonio Rodríguez Martín, Lídia Dionísio, Raquel Campos-Herrera

**Affiliations:** 1 UDIT MED—Mediterranean Institute for Agriculture, Environment and Development, Pólo, Universidade do Algarve, Campus de Gambelas, Ed 8, 8005-139 Faro, Portugal; fbuenopallero@gmail.com (F.Á.B.-P.); lpcdio@gmail.com (L.D.); 2 Departamento de Viticultura, Instituto de Ciencias de la Vid y del Vino (Gobierno de La Rioja, CSIC, Universidad de La Rioja), Finca La Grajera, Ctra. de Burgos Km. 6, 26007 Logroño, Spain; ruben.blanco@icvv.es (R.B.-P.); ignacio.vicente@icvv.es (I.V.-D.); 3 Departamento de Medioambiente, Instituto Nacional de Investigación y Tecnología Agraria y Alimentaria INIA, Ctra. de la Coruña, km 7.5, 28040 Madrid, Spain; rmartin@inia.es; 4 Departamento de Ciencias Biológicas e Biotecnologia, Faculdade de Ciências e Tecnologia, Universidade do Algarve, Campus de Gambelas, Ed 8, 8005-139 Faro, Portugal

**Keywords:** biologic control, entomopathogenic organisms, habitat preference, Mediterranean agro-ecosystems, multivariate analysis

## Abstract

Entomopathogenic fungi (EPF) are distributed in natural and agricultural soils worldwide. To investigate EPF occurrence in different botanical habitats and soil-ecoregions, we surveyed 50 georeferenced localities in the spring of 2016 across the Algarve region (South Portugal). Additionally, we compared three EPF isolation methods: insect baiting in untreated or pre-dried-soil and soil dilution plating on a selective medium. We hypothesized that forest habitats (oak and pine semi-natural areas) and the acidic soil ecoregion may favor EPF occurrence. Overall, EPF species were present in 68% of sites, widely distributed throughout the Algarve. The use of selective media resulted in higher recovery of EPF than did either soil-baiting method. Contrary to our hypothesis, neither vegetation type nor ecoregion appeared to influence EPF occurrence. Traditional and molecular methods confirmed the presence of five EPF species. *Beauveria bassiana* (34% of sites), was the most frequently detected EPF, using pre-dried soil baiting and soil dilution methods. However, baiting untreated soil recovered *Fusarium solani* more frequently (26% of sites), demonstrating the utility of using multiple isolation methods. We also found *Fusarium oxysporum, Purpureocillium lilacinum* and *Metarhizium anisopliae* in 14%, 8% and 2% of the sites, respectively. Three abiotic variables (pH, soil organic matter and Mg) explained 96% of the variability of the entomopathogen community (EPF and entomopathogenic nematodes) in a canonical correspondence analysis, confirming the congruence of the soil properties that drive the assemblage of both entomopathogens. This study expands the knowledge of EPF distribution in natural and cultivated Mediterranean habitats.

## 1. Introduction

Fungi are the predominant natural pathogens of arthropods [1]. Hypocrealean entomopathogenic fungi (EPF), such as those in the genera *Beauveria* and *Metarhizium*, are natural inhabitants of most terrestrial ecosystems, including natural and agricultural areas [2,3,4]. EPF can interact with arthropod hosts as parasites or saprophytes [5]. During the parasitic phase, after the conidia infect the host, the fungus produces various compounds responsible for host death [6,7] and other secondary metabolites with an antibiotic or antagonistic response to defend the cadaver from opportunistic organisms [5,8,9]. During the saprophytic phase, mycelia invade the entire body cavity to finally generate emergent mycelia and conidiophores for passive dispersion of the spores [10,11].

Among different methods to isolate EPF from the soil is the traditional insect-bait using *Galleria mellonella* (Lepidoptera: Pyralidae) as a host [12,13,14]. With the expectation of recovering additional EPF species [15], some studies have employed other insects such as *Tenebrio mollitor* (Coleoptera, Tenebrionidae) or combination of different hosts [16,17,18,19]. Other studies have used soil dilution and selective media to isolate EPF from soil [20,21]. Although alternative selective media recipes that lack dodine (N–dodecylguanidinemonoacetate) have been suggested [22], this fungicide, in combination with other antibiotics, is commonly used to isolate EPF and minimize contamination [21,23,24,25]. Combinations of insect-baits and selective media procedures are recommended to capture a wider range of EPF species [26]. Once isolated, fungal classification is often based on morphologic/morphometrical examinations that can be inaccurate when resolving closely related species. However, molecular tools are increasingly available for identification [21,27,28].

As natural regulators of arthropod populations in the ecosystem [29], EPF are well-known biologic control agents against a broad variety of arthropod pests [1,30,31,32]. Active research has developed commercial products based on EPF for decades—including mycoinsecticides derived from their active metabolites [9,29,33,34]. However, abiotic factors and biotic interactions that occur in the soil can affect their performance as biocontrol agents [1,34,35,36]. For example, soil granulometry can affect EPF communities: the greater porosity of sandy soils enhances the movement of fungal propagules, while clay substrates cause adsorption of conidia [36,37]. Similarly, the soil organic matter (SOM) content modulates EPF diversity by promoting the presence of insect hosts, while favoring some EPF antagonists [38]. Temperature and moisture are also key drivers of EPF presence and activity in soils [36].

Many soil organisms interact with EPF and vice versa [36]. Some microorganisms produce secondary metabolites that are toxic to EPF or inhibit their germination and growth [11,36]. EPF are weak saprophytes in natural conditions [1] and resource competition from other entomopathogens, such as the entomopathogenic nematodes (EPNs), may also restrict their occurrence. Several combined application studies using EPN and EPF reported that their interactions are not only species dependent (including the target host), but different concentrations and timing of application (simultaneous or sequential) are factors that can alter the outcomes [39,40,41]. Interactions of a similar kind occur among other microorganisms in the soil, such as nematophagous fungi [11]. EPF also establish complex and highly differentiated interactions with soil macroinvertebrates that can enhance or reduce EPF occurrence, such as earthworms which favor EPF dissemination and activity or collembolans that feed on them [36,42].

Habitats synthesize a combination of diverse biotic and abiotic properties and have been proposed as one of the main drivers for the EPF natural occurrence. *Beauveria bassiana* was reported linked with shaded semi-natural areas (oaks or pines) and *Metarhizium anisopliae* with crops soils [3,18,43]. However, *B. bassiana* is also a dominant EPF species in many Mediterranean agricultural areas [19,44,45]. Both natural and agricultural areas have been explored for the presence of EPF species in Spain [35,43,45]. Conversely, in Portugal, little is known in this regard except for a few surveys mainly focused on perennial crops such as olives and grapes [19,44]. Four main botanical groups characterize the Algarve region (South Portugal): oak (some still under cork production), pine (native populations or replanted areas), palmetto (wild areas) and citrus (the main agricultural sector in the region). This region also comprises two main soil-ecoregions: “calcareous” or “Barrocal” (predominantly basic soil with a high percentage of carbonates, poor in Fe and mainly located in the low-inland areas) and “non-calcareous” or “Serra and Littoral” (more acidic soils, not limited in Fe availability and mainly surrounding the Barrocal). Campos-Herrera et al. [46] investigated the natural occurrence of EPNs in the Algarve region and concluded that botanical group and some abiotic factors (particularly soil pH and clay content), appeared to modulate communities of EPNs and associated microorganisms. Herein, we extend that study to include the natural occurrence of EPF species. We speculated that vegetation type will drive EPF occurrence and activity, being especially favored in oak and pine semi-natural habitats [3,43] and that the dominant species will be *B. bassiana* as shown in previous studies in Mediterranean areas [19,45]. Based on the general premise that fungi tolerate acidic soils better than basic soils [47], we also hypothesized that non-calcareous soils would favor the presence of EPF. Finally, we predicted that those soil properties associated with the EPN soil food web assemblage [46] would also define the EPF community. We also expect different methods of EPF isolation to detect different patterns of recovery occurrence, activity and biodiversity [26]. Hence, this study aimed to explore ecological drivers of EPF species in the Algarve and to identify abiotic factors associated with their natural occurrence, based on the results provided by three isolation methods: untreated soil bait, pre-dried soil bait and soil dilution and culture in selective media. Thus, the specific objectives of this study were: (1) compare the natural EPF distribution and species composition derived from the three methods of isolation, (2) investigate the effect habitat type and soil-ecoregion on EPF distribution and species composition and (3) discriminate the abiotic factors that drive EPF and EPN assemblages

## 2. Materials and Methods

### 2.1. Study Area, Sampling Method and Soil Parameters

**Figure 1:**
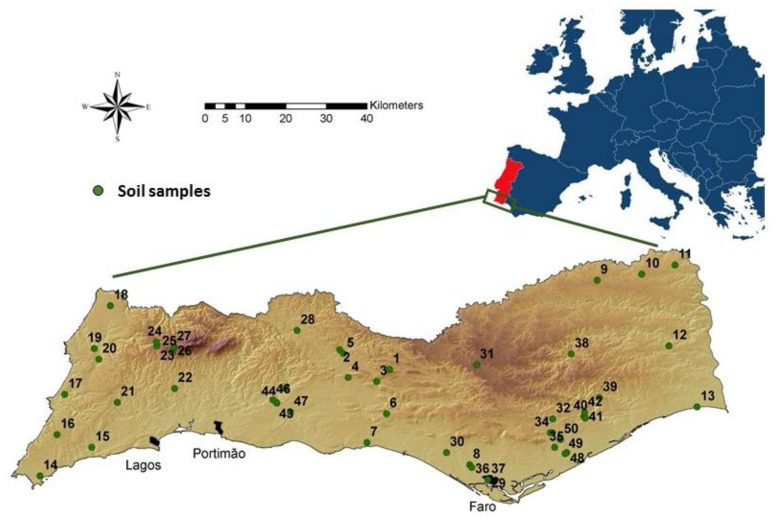
Distribution of the sampling sites along the Algarve region, Southern Portugal (see Campos-Herrera et al. [46] for additional details).

A total of 50 geographical localities distributed throughout the south coast and the interior of Portugal in the Algarve region (Southwest of continental Europe) were surveyed during spring 2016 (Figure 1). These localities represented four of the most widespread habitats in the region driven by the following botanical groups: oak (*Quercus* spp., Fagales, Fagaceae, n = 14), pine (*Pinus* spp. Pinales, Pinaceae, n = 14), palmetto (Arecales, Arecaceae, n = 7) and citrus orchard (*Citrus* spp., Sapindales, Rutaceae, n = 15). These localities were also assigned to one of the two typical soil-driven ecoregions knowns as “Barrocal” (n = 20) and “Serra and Littoral (n = 30), characterized as “calcareous” (alkaline soils with low Fe content) and “non-calcareous” (lower pH and higher Fe content). Campos-Herrera et al. [46] reported the details for geographical coordinates, localities and sampling schemes. Briefly, we collected two composite samples per locality in an area ∼0.5 ha, each comprised of 20 single soil cores (2.5 cm ø × 20 cm depth) and store them at 4 °C in dark conditions until further processing (<5 days). Hereafter, we well mixed all the cores of each samples in the laboratory and divided them into subsamples of 200 g of fresh soil employed for subsequent analyses. Campos-Herrera et al. [46] reported the soil parameters (pH, electric conductivity, organic matter, macro- and micronutrient elements and granulometry) analyzed by the Laboratório de Análises Químicas, LAQ (Universidade do Algarve, Faro, Portugal).

### 2.2. Entomopathogenic Fungi Isolation

We examined the EPF occurrence in all the samples (n = 100) by using three types of soil processing in each one to ensure a balanced analysis: (i) untreated soil, (ii) pre-dried soil and (iii) soil dilution and culture in selective medium. Hence, from each sample, we had three type of measurements of EPF natural occurrence. Overall, we baited the untreated and pre-dried soil samples following Zimmerman [12] and Meyling [13] procedures. First, the untreated soil samples employed in this study were the same as described by Campos-Herrera et al. [46], but separating the dead insect larvae which confirmed nematode emergences from those with signs of fungal death [21,48]. Second, the pre-dried soil samples were first lightly dried at 35 °C over a week to avoid any EPN infestation [43], then remoistened to half field capacity with distilled water [12,13]. For both bait tests, we used the final instar larva of *G. mellonella* as host, reared at Universidade do Algarve (Faro, Portugal), by performing two independent rounds of 10 larvae each per sample. Then, we incubated them for four days at 22–24 °C in dark conditions, inverting the containers daily to ensure the movement of the larvae through the soil regularly. Following Quesada-Moraga et al. [43], after assessing larval mortality, we disinfected each dead larva with 1–2% sodium hypochlorite solution for 3 minutes (rinsing with sterile distilled water between washes) and subsequently placed them into independent moist chambers with sterilized filter papers (RH > 90%, 27 ± 1 °C in dark conditions). Live larvae were incubated in the same experimental conditions for an additional 72 h to record possible late mortality. We revised the insect cadavers every 2–3 days during 16–20 days for the detection of fungal mycelium growth. Cadavers that showed abundant emerging mycelium (i.e., from intersegmental regions and natural holes) were considered as likely infected by EPF, while the presence of little to no mycelium on the surfaces of cadavers was associated with saprophytes and these were discarded [35].

For the third method of EPF isolation, we followed the procedure adapted by Shin et al. [23] and Korosi et al. [26]. The selective media was prepared with potato dextrose agar (PDA, Biokar, Lardero, La Rioja, Spain) supplemented with 0.1 g/L dodine (Sigma Aldrich, San Luis, MO, USA) and 0.1 g/L chloramphenicol (Sigma Aldrich) [23,26]. Briefly, five grams of dried soil from each subsample was suspended in Falcon® tube with 45 mL sterile half-strength Ringer solution and 0.05% Tween 80 [49]. The soil suspension was homogenized (Vortex^®^, Darmstadt, Germany) for 3 min. Preliminary tests on serial dilutions confirmed satisfactory results in terms of the facility to identify and remove individual fungal colonies from the plates when plating 100 µL of 10° suspension. We prepared 3 plates with selective media per sample. Thereafter, the plates were sealed with Parafilm^®^ and maintained at 27 °C in the dark until fungal growth was observed (7–10 days). Finally, we estimated the number of putative EPF colonies forming units (CFUs) and richness per plate.

A pure fungal culture was established in PDA from each cadaver with evidence of EPF growth derived from the untreated or pre-dried soil test as well as from the CFU retrieved from the selective media. To identify the causal agent of the death, we evaluated the pathogenicity of those pure cultures by confirming the Koch’s postulates. We placed three last instar *G. mellonella* into Petri dishes with PDA and actively growing mycelia (2 h, 27 ± 1 °C in dark conditions). We prepared two plates per isolate and another two control PDA plates for the control. Then, we placed the larvae independently in new Petri dishes with moistened study filters to incubate them in a humid chamber (RH > 90%, 27 ± 1 °C in dark conditions). After three days of larvae–fungus contact, we evaluated the larval mortality daily for a total of 8 days, processing the insect cadavers following the disinfecting and cleaning protocol previously described for the baiting methods (adapted protocol from Cabrera-Mora et al. [50]). During this period, larvae did not receive any supplemented food. To ensure that the larvae were healthy, not dying because of starvation and the experimental conditions were appropriate, we verified that the surviving larvae pupated and emerged as adults.

### 2.3. Fungal Identification

All fungi that confirmed Koch’s postulates were considered EPF. For their identification, we first performed a preliminary macro-/microscopical description using taxonomic keys based on morphologic characteristics [51,52,53]. Semi-permanent slide mounts of lactophenol cotton blue were prepared and observed in the microscope. Pictures of representative species were also recorded. We recovered the mycelium of the selected fungi for the establishment of an EPF collection and to store at −20 °C for further molecular identification. For DNA extraction, we first mechanically disaggregated the mycelia for 15 seconds by using a sterile blue pestle assembled to a pellet mixer (VWR International, Lutterworth, UK). Then, we followed the maximum yield protocol of the Speedtools tissue DNA extraction kit (Biotools, Madrid, Spain). DNA of each sample was eluted in 50 μL of Milli-Q water (Milli-Q Water Systems, Millipore S.A., Molsheim, France), analyzed for quality and quantity (nanodrop system, Thermo Scientific 200 °C spectrophotometer) and stored at–20 °C until subsequent analysis.

By using the universal primers ITS1 and ITS4 [54], we evaluated the ITS region (ITS1, 5.8S and ITS2) as barcoding for the molecular identification of the selected fungi [19]. For ensuring optimal amplification in conventional PCR, all the DNA samples were previously diluted to a range between 0.5–1 ng/μL. Each reaction was performed in a final volume of 20 μL and included 1 μL DNA template, 200 nM dNTP mix (Promega, provided by Promega Biotech Iberica SL, Alcobendas, Madrid, Spain), 1× PCR buffer (5× ColorlessGoTaq® Reaction buffer, Promega), 400 nM of each primer (Biosearch Technologies, supplied by Biotools, Spain) with 0.68 U GoTaq^®^ G2 DNA Polymerase (Promega). Amplifications procedures were performed as described by Luo and Mitchell [55], employing the thermocycler Biometra T gradient (Biolabs, France). Each PCR product was verified for expected size by visualization after electrophoresis in 2% agarose gel in TAE (pH 8.3 ± 0.1) (Fisher Bioreagents, Ltd., Madrid, Spain) run along the reference BenchTop 100 bp DNA ladder (Promega). Individual bands were purified by QIAquick Gel Extraction (Qiagen^®^, Hilden, Germany) and the occurrence of the expected band verified in a TAE 0.8% agarose gel. All the samples were sequenced at Macrogen (Macrogen Europe Laboratory, Inc., Madrid, Spain). For each sample, the sequences (forward and reverse) were assembled (Geneious, R.5.6.5., Biomatters, Inc., Auckland, New Zealand), compared to reported sequences using Blast (http://blast.ncbi.nlm.nih.gov) and submitted to Genbank.

### 2.4. Comparison of the Detection Methods and Identification of Ecological Drivers for the Entomopathogenic Fungi Natural Occurrence

For each EPF isolation method, we included all fungal isolates that confirmed Koch’s postulates for the estimation of the frequency of occurrence (positive sites per total sites, expressed as percentage). For the insect baits methods, we also analyzed the larval mortality percentage. After examining for possible correlations (Pearson test) among the results obtained by different methods, we analyzed whether these variables were statistically significant among those methodologies by using one-way ANOVA and Tukey’s HSD test (*p* < 0.05) performed with SPSS 25.0 (SPSS Statistics, SPSS, Inc., Chicago, IL, USA). We also estimate the percentage of total EPF occurrence by combining the data obtained for all three EPF isolation methods [19,26,49]. For this combined data, we counted a site as positive for the EPF detection independently that resulted positive in one, two or the three methods and if one or more species were detected. Moreover, to provide additional data on the virulence of the isolates, we calculated the average time the isolates derived from each detection method took to kill the larvae in Koch’s postulate tests for all isolation methods. We visualized in maps the regional data derived from (i) the EPF occurrence values depending on the isolation method, (ii) the total EPF presence/absence and (iii) species isolated per site by using the SPSS statistical package for Windows V19 and ArcGis 10.

Additionally, we analyzed the impact of the botanical habitats (oak, pine, palmetto and citrus grove) and soil-ecoregions (calcareous and non-calcareous) on the percentages of EPF occurrence and compared the results obtained among the three isolation methods. We assessed significant differences among habitat types or ecoregions, as well as among the isolation method by using one-way ANOVA and Tukey’s HSD test and *t-*test (*p* < 0.05) performed with SPSS 25.0. Before statistical analysis, all the variables were arcsine transformed. We used least-square means ± S.E. as descriptive statistics.

### 2.5. Relationships of Entomopathogenic Fungi within Abiotic Factors and Multivariate Analysis of the Entomopathogen Community Assemblage

First, we established the range of the abiotic variables for the natural occurrence of each EPF species identified in this survey. Then, multivariate analyses of the selected entomopathogen organisms and environmental factors were performed using CANOCO 5 [56,57]. We employed the eight abiotic variables selected by Campos-Herrera et al. [46] to avoid strong co-linearity: elevation and the soil properties of pH and clay, OM, P, Mg, Zn and Fe content, to be included as explanatory (predictors) variables in CANOCO. For the dependent (response) variables, we selected target species that were present in at least 10% of the field sites [58]. We explored the assemblage of two kinds of soil entomopathogen organisms: fungi and nematodes. We retrieved the EPN inputs from the dataset presented by Campos-Herrera et al. [46], while for EPF we used the total occurrence (the combination of the numbers obtained for the three methods) of each fungal species. Prior to analysis, both biotic and abiotic variables were standardized by dividing by the highest values, ranking all values 0–1 [59]. As described by Campos-Herrera et al. [46], Detrended Canonical Correspondence Analysis (DCCA) was used to estimate the length of the system, selecting a Canonical Correspondence Analysis (CCA, constrained axes) when heterogeneous communities were detected [56]. We used the CCA (interspecies correlations) with Monte Carlo permutation (*n* = 499) and automatic forward selection for the assignment of significant environmental variables, using the *p*-values derived from the forward selection. The graphical results of the CCA were presented with bi–plot scaling (CANOCO 5).

## 3. Results

### 3.1. Distribution of Entomopathogenic Fungi across the Algarve Region: Comparison of Three Methods of Isolation

**Figure 2:**
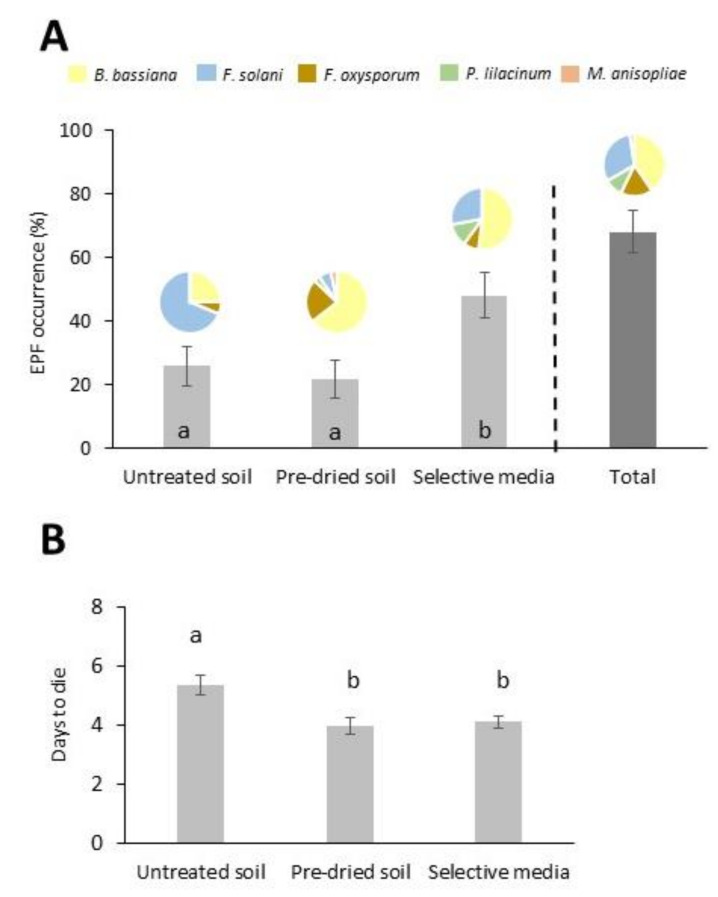
Entomopathogenic fungi (EPF) recorded by using three isolation methods and total EPF recovery (data from all methods combined) (**A**) Recovery frequency in the overall survey. EPF species averages are proportionally represented in pies; (**B)** number of days to kill *Galleria mellonella* larvae by the isolates derived from each of the isolation methods. Data derived from pure cultures in PDA and evaluated by Koch’s postulates test. Different letters indicate significant differences (*p* < 0.05) in Tukey’s test (HSD). Values are least-square means ± SE.

**Figure 3:**
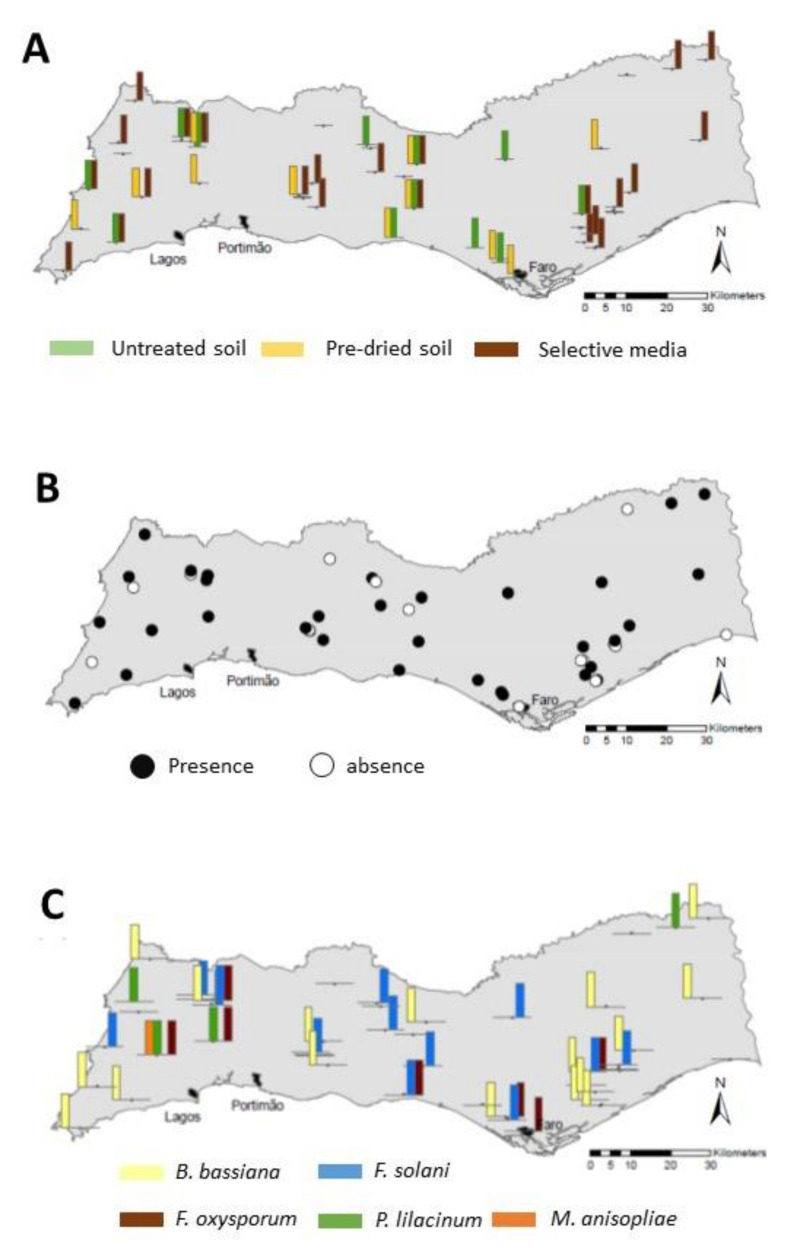
Geographical distribution for entomopathogenic fungi (EPF) occurrence in the region of Algarve (Southern Portugal) (**A**) Positive sites for each isolation method; (**B**) positive sites for any isolation method; (**C**) distribution of EPF species.

The EPF occurrence detected by the selective medium method were significantly higher than those reported through bait methods (F_2,147_ = 4.70, *p* = 0.011; Figure 2A). The average larval mortality percentage detected by the untreated soil bait was 0.9% ± 0.26 and by the pre-dried 1.6% ± 0.70 and differences between methods were not significant. The isolates cultured in PDA and derived from the untreated soil method resulted in a significantly longer time to kill *G. mellonella* larvae than those from the pre-dried soil bait and selective medium (F_2,74_ = 6.44, *p* = 0.003; Figure 2B). The results for the isolation methods were not significantly correlated, neither the EPF occurrence (the three methods) nor the larval mortality (only the two bait methods) (Supplementary Materials, Table S1). In addition, we found certain geographical differences for the EPF detection efficiency among isolation methods, as shown by the almost exclusive EPF detection in the eastern areas of the Algarve region by the selective medium method (Figure 3A).

Overall, 68% of the sites, widespread all along the sampling area, were positive for the occurrence of any EPF species (Figure 2A, Figure 3B). Five fungal species that confirmed Koch’s postulates were identified by morphologic and molecular identifications (Table 1). The species *Beauveria bassiana* and *Fusarium solani* were the most prevalent (Table 1) and widely distributed EPF (Figure 3C), detected in 34% and 26% of the sites, respectively. The distribution of *F. oxysporum*, present in 14% of the sites (Table 1), was mainly restricted to the central area of the Algarve region (Figure 3C). All the isolation methods identified those three abundant fungal species (Table 1; Figure 2A) with similar efficiency of recovery (Supplementary Materials, Table S2). Conversely, *Purpureocillium lilacinum* was reported only in four sites (none in untreated soil baits), mainly located in the northwest of the Algarve (Figure 3C) and its detection slightly differed among methods (Table S2). Finally, we detected the species *Metarhizium anisopliae* in just one site using the pre-dried soil (Table 1, Figure 3C). In summary, the pattern of EPF occurrence by species was different among methods (Figure 2A). We detected all five EPF species only through pre-dried soil baits, but only three species in untreated soil baits. Accordingly, the method of isolation, *F. oxysporum* was the dominant species in untreated soil baits, while *B. bassiana* resulted the dominant in pre-dried soil baits and selective media method (Figure 2B).

### 3.2. Impact of Ecological Drivers on Entomopathogenic Fungi Natural Occurrence

**Figure 4:**
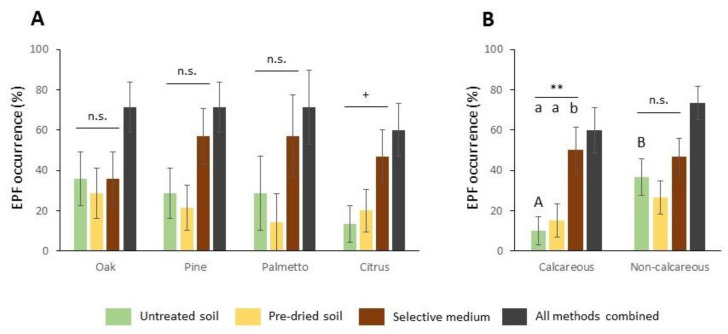
Comparison of entomopathogenic fungi (EPF) recovery frequency using different isolation methods depending on two ecological drivers (**A**) botanical habitats; (**B**) soil ecoregion. Asterisks indicate significant differences within treatment comparisons at ** *p* < 0.01, ^+^ *p* < 0.1 and n.s., not significant. Different letters indicate significant differences in Tukey’s test (HSD) (*p* < 0.05). Values are least-square means ± SE.

We obtained similar ecological trends independently of the EPF isolation method employed, except for higher EPF occurrence using untreated soil baits in non-calcareous soils (Figure 4B; Supplementary Materials, Table S3). EPF occurrence was also similar for all isolation methodologies except in calcareous soils, where the EPF recovery was significantly higher using the selective medium method (Figure 4B; Supplementary Materials, Table S4). Larval mortality percentage was not significantly different between soil bait methods regardless of botanical group or ecoregion. The number of colonies and richness per sample observed by the selective medium method did not differ among habitat types or ecoregions. We found the species *B. bassiana* and *F. oxysporum* in similar percentages (Table S2) in all botanical groups and both soil ecoregions (Supplementary Materials, Figure S1). Only *P. lilacinus* showed higher recoveries in non-calcareous soils than in calcareous (Figure S1; Table S2), while *M. anisopliae* was only detected once, in the semi-natural oak habitat of non-calcareous soils.

### 3.3. Abiotic Ranges for the Occurrence of Entomopathogenic Fungi and Patterns of Assemblage into Selected Members of the Entomopathogenic Community

Overall, EPF occurred broadly among all the soil fertility parameters (Table 1). The three most abundant fungal species, *B. bassiana*, *F. solani* and *F. oxysporum* were detected in all botanical groups (except *F. oxysporium* not encountered in palmetto habitats) and both soil ecoregions, from sea level up to 500 m ASL and for wide ranges of soil pH and granulometry. The species *P. lilacinus*, only reported in 8% of the sites, was present in oak and pine habitats and non-calcareous soils, with loam/clay loam texture, lightly acidic soils and high SOM and Fe content. The only positive site for *M. anisopliae* was located in the west of the Algarve region at high altitude (around 100 m ASL), in oak habitat and non-calcareous soils with clay loam texture, lightly acidic soils and high SOM and Fe content.

**Figure 5:**
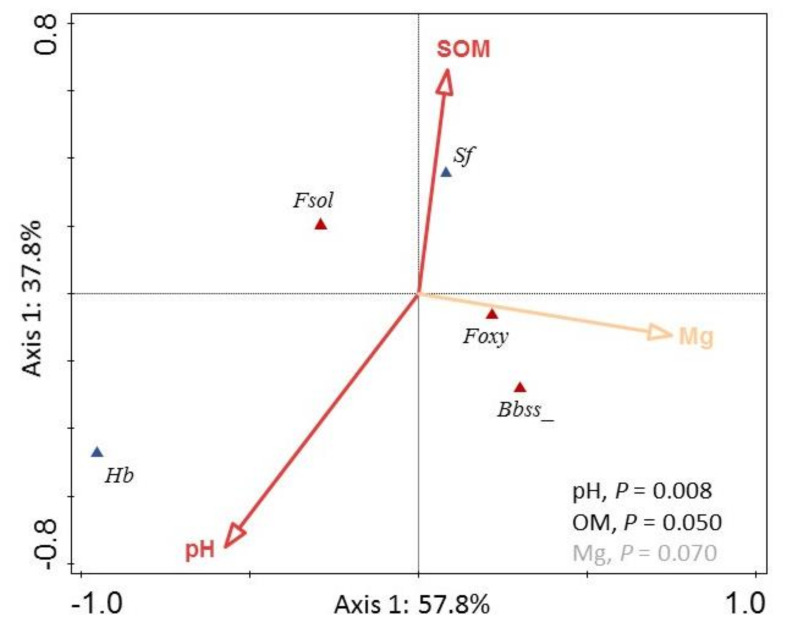
Canonical correspondence analysis depicting the regional distribution and relationships between significant abiotic factors and soil organisms (*p* < 0.05 in red and *p* < 0.1 in orange) in the Algarve region. Codes for abiotic factors (arrows): soil organic matter (SOM), soil pH (pH) and Mg content (Mg). Codes for biotic factors (triangles): the entomopathogenic fungi species (brown triangles) *Beauveria bassiana* (Bbss), *Fusarium solani* (Fsol) and *F. oxysporum* (Foxy); and the entomopathogenic nematodes (blue triangles) *Heterorhabditis bacteriophora* (Hb) and *Steinernema feltiae* (Sf).

We selected eight abiotic factors (elevation, soil pH and clay, SOM, P, Mg, Zn and Fe content) as explanatory variables (see Campos-Herrera et al., [46] for more details) in the multivariate analysis of the soil entomopathogen species present in at least 10% of the sites (three EPF and two EPNs). CCA was conducted since DCCA maximum length was 4.1. The first two axes accounted for 95.6% of the explained fitted variation in species–environment relationships (Figure 5). The explanatory variables SOM and soil pH significantly contributed to explaining the biotic assemblage (*p* < 0.05), defining mainly the axis 2, while Mg content marginally contributed (*p* = 0.07) and slightly defined axes 1. Overall, the EPF species were more associated with the axis 1, with *B. bassiana* and *F. oxysporum* defined by high Mg concentrations and hence, associated with calcareous soils, but *F. solani* showing the opposite association. By contrast, the contrary, the EPN species were more associated with axis 2: *H. bacteriophora* with high soil pH and *S. feltiae* with high SOM.

## 4. Discussion

### 4.1. Distribution of Entomopathogenic Fungi across the Algarve Region and Comparison of Three Isolation Methods

Overall, 68% of the sites were positive for the presence of EPF species in the Algarve region. Previous surveys found similar percentages, for example, 52% in the Pacific Northwest [60] and 72% in Spain [43]. However, EPF occurrence was quite variable in other studies, with only 20–30% of detections reported in the UK, Mexico, Turkey and Tasmania [16,61,62,63] or over 90% in Ontario (Canada) and Switzerland [2,64]. Those frequencies are difficult to compare due to the lack of methodological uniformity among surveys. Not all the studies made the same effort in recovering the fungi. While Quesada-Moraga et al. [43] baited 50 *G. mellonella* larvae per site in five independent soil subsamples, Chandler et al. [62] employed just one larva per sample. In our study, by using three different EPF isolation methods and employing 20 *G. mellonella* larvae per sample in two independent baiting rounds, we increased the likelihood of recovering different entomopathogens [65], including those of distinct guilds (EPF and EPNs) in the same sample [46]. Similarly, it is possible that increasing the size of the samples and the number of samples per site and repeating the study at various times [3], we could have detected a higher incidence of EPF in the Algarve region. However, we collected all samples in springtime, which may be the most favorable season for surveying EPF in Mediterranean habitats [44,45] and hence, the overall picture of their natural occurrence can be detected. In any case, further studies including other habitat types and seasonal pattern can provide new insights on the EPF natural distribution in Algarve and other Mediterranean areas.

As we hypothesized, we observed different patterns of EPF occurrence by using different isolation methods, particularly between insect baits and the selective medium methodology. Although the serial soil dilutions plating on selective media is considered a quantitative method and insect baits provide semi-quantitative data [36], we analyzed variables that allowed comparisons among the three methodologies. Although selective media recover pathogenic and saprophytic phases of EPF [13], we resolved this methodological restriction by confirming the pathogenic capability of the isolates via Koch´s postulates. Two independent rounds in soil baits allowed the isolation of EPF that remained inactive in the first round, as recommended for retrieving inactive EPNs [65]. Despite these protocols, the EPF occurrence and larval mortality detected by both soil baits were significantly lower than found using selective media. Especially low EPF occurrence and pathogenicity rates occurred using untreated soil baits, suggesting that antagonism or competition with other entomopathogens such as EPNs could interfere with efficient EPF isolation compared with pre-dried baits [46]. Additionally, the incubation of the soil bait samples at different temperatures could increase the recovery of different EPF species and provide differential patterns of distribution [66]. In any case, no correlations among methodologies were established for any of the variables studied (as observed by Kessler et al. [67]), suggesting that each method may be detecting EPF in different active stages [36]. Consequently, the combined results of all methodologies likely provided a more realistic distribution of EPF in the Algarve region [26].

### 4.2. Entomopathogenic Fungi Species Composition and the Ecological Drivers of Their Natural Occurrence

Our detection of five EPF species is similar to previous studies: four in Spain [35], six in Turkey [63] and in Egypt [68] and seven in Denmark [3]. However, some studies also reported a higher or lower number of isolates, such as 8 species in olive groves in Portugal [44], 9 in agricultural areas in Spain [45], 12 in Portuguese vineyard soils [19] or the only two species (*B. bassiana* and *M. anisopliae*) in Spain and Mexico [16,43], respectively. It is noteworthy that Campos-Herrera et al. [46] employed the same untreated soil samples for evaluating the presence of EPNs, so the higher abundance of *F. solani* found through this particular methodology may be related to its interaction with EPNs. For example, Wu et al. [69] observed that *F. solani* increased the efficacy of the EPN species *Steinernema diaprepesi.* Our observations could support the hypothesis that both entomopathogenic organisms cooperate in a mutual strategy for the control of insect pests, but additional studies are needed for confirmation. Finally, *P. lilacinum* was the only EPF species that showed significant differences among isolation methodologies, being favored by the selective medium method. These results highlight the necessity of using various isolation methods to unravel as much as possible the presence of different EPF species in soil communities.

In agreement with previous surveys conducted in the Iberian Peninsula [19,35,43,44,45], *B. bassiana*, identified in 34% of the sites, was the prevalent EPF species in Algarve’s soils. The following two most common fungal species were *F. solani* and *F. oxysporum*, present in 26% and 14% of the sites, respectively. In addition, the species *P. lilacinum* and *M. anisopliae*, isolated just in a few sites, were previously detected in low numbers in the Iberian Peninsula [44,45]. The EPF species *B. bassiana*, widespread in soils, is commonly used in commercial products [1]. Sharma et al. [19] also describe a *P. lilacinum* strain isolated from soils of Northern Portugal as an entomopathogen. Although *F. solani* and *F. oxysporium* are not frequently reported as entomopathogen organisms, there are some exceptions, such as the isolates from Egyptian soils of both fungal species described by El-Ghany et al. [68] using *G. mellonella* as bait. In addition, Wu et al. [69] isolated *F. solani* in *Diaprepes abbreviatus* (Coleoptera: Curculionidae) soil bait. Moreover, a recent review by Sharma and Marques [70] disentangled the complex nature of various *Fusarium* spp., observing opportunistic, saprobic and entomopathogenic behaviors. Indeed, *F. oxysporum* and *F. solani* are suggested to have insecticidal properties and the ability to develop inside hosts [70].

Concerning the ecological drivers and contrary to our hypothesis, botanical habitats did not affect EPF occurrences nor larval mortalities in soils of the Algarve region. Previous studies obtained contrasting results. Quesada-Moraga et al. [43], in agreement with our observations, did not find significant differences among habitats (natural *versus* agricultural) for the overall EPF presence, but observed species-specific habitat preferences. On the contrary, Sánchez-Peña et al. [16] observed overall higher EPF occurrence in oak areas (51% of the sites) than other botanical habitats (less than 18% of the sites in pine, chaparral and agricultural areas). The high number of soil samples (280) analyzed by Sánchez-Peña et al. [16] may have favored the observation of statistical significance. Since fungi generally tolerate acidic soils better than basic soils [47], we expected higher EPF detections in the non-calcareous region. Indeed, the three predominant EPF species in the Algarve (*B. bassiana, F. solani* and, *F. oxysporum*) were retrieved in soils ranging from pH 5 to 8, but for the total fungal species isolated through all methodologies, this general trend was not supported. Optimal growth ranges may be relevant in determining the predominance of some species over others [43]. Providing information on optimal ranges for EPF occurrences could improve predictive models useful in biologic control strategies.

### 4.3. Entomopathogenic Fungi Assemblage Patterns

Many environmental factors can affect the EPF natural occurrence. Besides temperature and humidity, key factors for the EPF reproduction and activity [36], soil properties that provide the microhabitat conditions associated with the EPF are very important. Several authors have discussed how soil properties can affect the EPF natural occurrence [21,36,43,71,72]. The soil pH and soil organic matter content were the two soil properties that mainly explained the variability of the EPF and EPN species included in our multivariate linkages. These two variables were also reported as relevant factors in previous studies of similar nature [43,46,71,72]. The typical consideration that fungus tolerate acidic soils better than basic soils [47], was also shown to some extend with the EPF detected in Algarve. Despite some exceptions to the most detected species: *B. bassiana*, *F. solani* and *F. oxysporum,* results in line with the ecoregion pattern commented before, the multivariate analysis showed that the EPF species were mainly distributed in areas with acidic pH. However, previous studies in the Iberian Peninsula showed a higher detection in basic soils [43]. However, the fact that in this study the most prevalent species were *B. bassiana*, *F. solani* and *F. oxysporum* can explain the differences observed in the preferred pH recorded by Quesada-Moraga et al. [43], where only *B. bassiana* and *M. anisopliae* were recorded. In addition, low soil organic matter content is often associated with higher EPF occurrence [43,72]. It is noteworthy that different EPF and EPN assembled differentially, occupying different quadrants in the CCA analysis. Thus, while the EPF species *B. bassiana* and *F. oxysporum* were slightly linked to calcareous soils, *F. solani* was more associated with non-calcareous soils. Finally, EPNs followed a similar pattern that was observed by Campos-Herrera et al. [46] when other soil organisms, excluding EPF, were included in their CCA analyses.

## 5. Conclusions

This study expands the understanding of EPF natural distribution and increases the number of EPF species previously described in the Iberian Peninsula [19,35,43,44,45]. It also explores the ecological drivers that could modulate their occurrence in the Algarve region. The study provides a more comprehensive characterization of the EPF occurrence by using three different isolation methodologies, combining traditional and molecular tools for species identification. Moreover, the description of the EPF/EPN community assemblage by the soil properties provides new insights on shared niches among different guilds. Under a restrictive and prohibitive context of many agrochemical products, identifying the best ecological scenarios for the mutual use of different beneficial soil organisms [73,74], promoting cooperation and avoiding competition for hosts [11], will provide more effective bio-tools that can contribute to a more sustainable agriculture.

## Figures and Tables

**Table 1:** Summary of soil properties and general characteristics associated with entomopathogenic fungi (that confirmed Koch’s postulates) isolated in the Algarve (Portugal).

	Entomopathogenic Fungi Species				
Variables for Characterization	*Beauveria bassiana*	*Fusarium solani*	*Fusarium oxysporum*	*Purpureocillium lilacinum*	*Metarhizium anisopliae*
General characteristics					
Isolation method ^a^	U, D, S	U, D, S	U, D, S	D, S	D
No. positive sites	17 (34%)	13 (26%)	7 (14%)	4 (8%)	1 (2%)
GenBank accession number	MN808334	MN808329, MN808331	MN808330, MN808332	MN808335	MN808333
General properties					
Habitat type	oak, pine, palmetto, citrus	oak, pine, palmetto, citrus	oak, pine, citrus	oak, pine	oak
Ecoregion	calcareous, non-calcareous	calcareous, non-calcareous	calcareous, non-calcareous	non-calcareous	non-calcareous
Altitude (m ASL)	23–527	9–500	4–527	99–215	99
pH	5.0–8.1	4.9–8.1	5.1–8.0	5.1–6.1	6.1
CE (µS/cm)	65–523	82–430	65–402	66–374	374
SOM (%)	1.6–11.1	2.3–17.6	1.8–10.8	3.8–10.8	10.8
Sand (%)	25–87	25–80	25–80	26–47	33
Silt (%)	10–44	7–49	15–39	31–43	39
Clay (%)	3–48	3–37	5–39	18–35	28
Soil fertility					
P (mg·kg^−1^)	0.02–47.5	0.17–49.1	0.02–25.2	0.02–3.9	0.01
K (mg·kg^−1^)	23–110	25–165	25–141	25–127	127
Mg (mg·kg^−1^)	49–1160	4–1068	49–746	220–646	646
Ca (mg·kg^−1^)	529–3867	545–4851	542–2589	584–1510	1510
Zn (mg·kg^−1^)	0.04–7.22	0.03–9.60	0.06–4.73	0.90–3.10	3.11
Mn (mg·kg^−1^)	7.7–34.6	2.7–35.0	11.5–34.6	33.5–34.6	34.6
Fe (mg·kg^−1^)	3.8–76.7	0.9–67.9	3.3–62.1	40.3–73.7	40.3
Cu (mg·kg^−1^)	0.01–4.97	0.02–8.13	0.01–2.75	0.5–1.6	1.40

^a^ Method of isolation code: U—untreated soil; D—pre-dried soil; S—soil dilution and plating in selective medium. ^b^ Representative isolates deposited in the UAlg and ICVV collections.

## Supplementary Materials

The following are available online at https://www.mdpi.com/2075-4450/11/6/352/s1, Figure S1. Comparison of entomopathogenic fungi (EPF) recovery frequency by species depending on two ecological drivers. A. Botanical habitats. B. Soil ecoregion. Different letters indicate significant differences in T-test (*p* < 0.05). Values are least-square means ± SE, Table S1. Pearson correlations (*p* < 0.05) to compare the recovery occurrence and larval mortality percentages among different isolation methods of entomopathogenic fungi (EPF); n.s., no significant, Table S2. Statistical analysis (One-way ANOVA and T-test) for the occurrence of fungal that confirmed Koch´s postulates accordingly the variables isolation method, vegetation type, and soil eco-region, Table S3. Statistical analysis (One-way ANOVA and T-test) of the impact of the variables vegetation type or soil ecoregion on the occurrence of entomopathogenic fungi (EPF) and larval mortality recorded for each of EPF isolation method, Table S4. Statistical analysis (One-way ANOVA) of the efficiency among isolation methods for the occurrence of entomopathogenic fungi (EPF) and larval mortality recoveries efficacy depending on the factors vegetation type or ecoregion.
