# Supplementary material for: Patterns of Occurrence and Activity of Entomopathogenic Fungi in the Algarve (Portugal) Using Different Isolation Methods
Source: Insects. 2020 Jun 4;11(6):352. doi: 10.3390/insects11060352 (PMC7348715; doi:10.3390/insects11060352)
Supplement: Supplementary file 1 [file insects-11-00352-s001.pdf]

Table S1. Pearson correlations ($p < 0.05$) to compare the recovery occurrence and larval mortality percentages among different isolation methods of entomopathogenic fungi (EPF). Codes: n.s., no significant; n.d., no data.

	**Recovery Occurrence%**		
	**Fresh soil**	**Pre-Dried Soil**	**Selective Media**
Untreated soil bait	–	n.s.	n.s.
Pre-dried soil bait		–	n.s.
Selective medium			–
	Larval mortality%		
Untreated soil bait	–	n.s.	n.d.
Pre-dried soil bait		–-	n.d.
Selective medium			–

Table S2. Statistical analysis (One-way ANOVA and *t*-test, $p < 0.05$) for the occurrence of fungal that confirmed Koch´s postulates accordingly the variables isolation method, vegetation type and soil eco-region. Code: n.s., no significant.

EPF Species	EPN isolation Method F (*P*)	Vegetation Type F (*P*)	Ecoregion t (*P*)
B. bassiana	2.206 (n.s.)	0.402 (n.s.)	0.769 (n.s.)
F. solani	1.000 (n.s.)	0.544 (n.s.)	1.302 (n.s.)
F. oxysporum	2.478 (n.s.)	0.569 (n.s.)	0.478 (n.s.)
P. lilacinum	2.972 (0.054)	n.d.	n.d.
M. anisopliae	n.d.	n.d.	n.d.

Supplementary material 3

Table S3. Statistical analysis (One-way ANOVA and *t*-test, $p < 0.05$) of the impact of the variables vegetation type or soil ecoregion on the occurrence of entomopathogenic fungi (EPF) and larval mortality recorded for each of EPF isolation method. Codes: n.s., no significant; n.d., no data

EPF Isolation Method	EPF Occurrence%		Larval Mortality%	
	**Vegetation Type F (*P*)**	**Ecoregion t (*P*)**	**Vegetation Type F (*P*)**	**Ecoregion t (*P*)**
Untreated soil bait	0.862 (n.s.)	2.362 (0.022)	0.642 (n.s.)	2.527 (0.016)
Pre-dried soil bait	0.197 (n.s.)	1.006 (n.s.)	1.165 (n.s.)	1.799 (n.s.)
Selective medium	0.494 (n.s.)	0.226 (n.s.)	n.d.	n.d.
All methods combined	0.196 (n.s.)	0.958 (n.s.)	-	-

Supplementary material 4

Table S4. Statistical analysis (One-way ANOVA, $p < 0.05$) of the efficiency among isolation methods for the occurrence of entomopathogenic fungi (EPF) depending on the factors vegetation type or ecoregion. Code: n.s., no significant.

**Ecological Drivers**	**EPF Occurrence% F (*P*)**
Vegetation type	
Oaks	0.100 (n.s.)
Pines	2.256 (n.s.)
Palmetto	1.500 (n.s.)
Citrus	2.492 (n.s.)
Ecoregion	
Calcareous	5.791 (0.005)
No-calcareous	1.286 (n.s.)

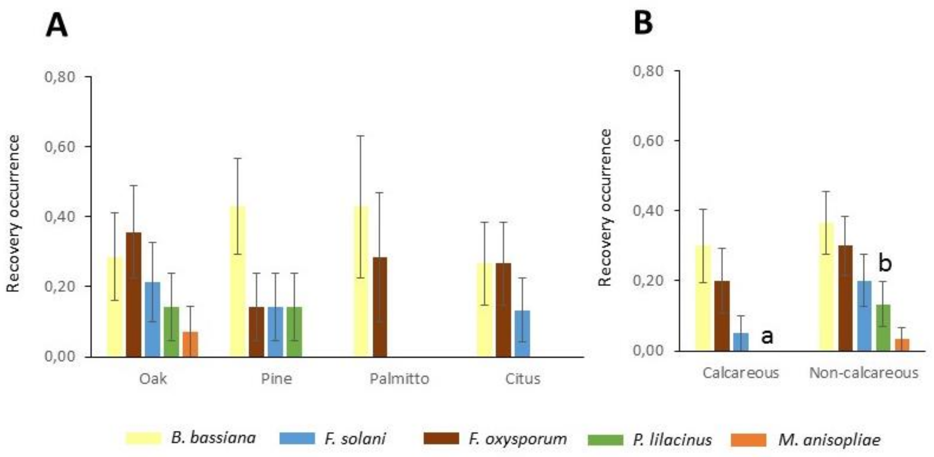

Figure S1. Comparison of entomopathogenic fungi (EPF) recovery frequency by species depending on two ecological drivers. **A.** Botanical habitats. **B**. Soil ecoregion. Different letters indicate significant differences in *t*-test ($P < 0.05$). Values are least-square means ± SE.
